# Supplementary material for: How Comorbidities Affect Hospitalization from Influenza in the Pediatric Population
Source: Int J Environ Res Public Health. 2022 Feb 28;19(5):2811. doi: 10.3390/ijerph19052811 (PMC8910429; doi:10.3390/ijerph19052811)
Supplement: Supplementary file 1 [file ijerph-19-02811-s001.zip › ijerph-1586858-supplementary.pdf]

Supplementary Table S1: Diagnoses and ICD-10 codes

Category	Diagnosis	ICD-10-code
**Heart disease**		
	Fenestrated atrial septum	Q20.0
	DORV with subaortic VSD	Q20.1
	Single ventricle	Q20.4
	VSD (ventricular septal defect)	Q21.0
	ASD (atrial septal defect)	Q21.1
	Congenital tricuspid atresia	Q22.4
	Tricuspid regurgitation, congenital	Q22.8
	Bicuspid aortic valve	Q23.1
	Congenital subaortic stenosis	Q24.4
	Congenital diverticulum of left ventricle	Q24.8
	Congenital malformation of heart	Q24.9
	PDA (patent ductus arteriosus)	Q25.0
	Right aortic arch and left descending aorta	Q25.47
	Capillary malformation	Q27.9
	Pulmonary hypertension	I27.20
	Secondary pulmonary arterial hypertension	I27.21
	Pulmonary stenosis, valvar	I37.0
	Hypertrophic cardiomyopathy	I42.2
	Cardiomyopathy	I42.9
	Hypertrophic cardiomyopathy secondary to Friedreich's ataxia	I43
	SVT (supraventricular tachycardia)	I47.1
	PAC (premature atrial contraction)	I49.1
	Benign atrial arrhythmia	I49.9
	Chronic combined systolic and diastolic heart failure	I50.42
	CHF (congestive heart failure)	I50.9
	Ascending aorta dilatation	I77.810
**Malignancy**		
	Acute lymphoid leukemia	C91.00
	ALL (acute lymphoid leukemia) in remission	C91.01
	Acute myeloid leukemia	C92.00
	Acute myeloid leukemia in remission	C92.01
	Myeloid leukemia associated with Down syndrome	C92.90
	Leukemia	C95.90
	T lymphoblastic lymphoma	C83.50
	Burkitt lymphoma	C83.70
	Lymphoma	C85.90
	Malignant neoplasm of left calcaneus	C40.32
	Pilocytic astrocytoma of cerebellum	C71.6
	Cancer	C80.1

	Brain tumor	D49.6
	History of chemotherapy	Z92.21
**Endocrine and Metabolic disorder**		
	Albinism	E70.30
	Oculocutaneous albinism	E70.329
	Hyperammonemia	E72.20
	Nonketotic hyperglycinemia	E72.51
	Lactose intolerance	E73.9
	Congenital disorder of glycosylation associated with mutation in PMM2 gene	E74.8
	Hypercholesterolemia	E78.00
	Hypertriglyceridemia	E78.1
	Hyperbilirubinemia	E80.6
	Hyperphosphatemia	E83.39
	Dehydration	E86.0
	Fluid deficit	E86.9
	Dehydration with hypernatremia	E87.0
	Hyponatremia	E87.1
	Lactic acidosis	E87.2
	Hypokalemia	E87.6
	Electrolyte and fluid disorder	E87.8
	Hypoalbuminemia	E88.09
	Mitochondrial disease	E88.40
	Ketosis	E88.89
	Central hypothyroidism	E03.8
	Hypothyroid	E03.9
	Hashimoto's disease	E06.3
	Ketotic hypoglycemia	E16.1
	Hypoglycemia	E16.2
	SIADH (syndrome of inappropriate ADH production)	E22.2
	Panhypopituitarism	E23.0
	Diabetes insipidus	E23.2
	Adrenal crisis	E27.2
	Adrenal insufficiency	E27.40
	Secondary adrenal insufficiency	E27.49
	Adrenal mass, left	E27.8
Diabetes	Diabetic ketoacidosis without coma associated with type 1 diabetes mellitus	E10.10
	Diabetes mellitus type 1, uncontrolled, without complications	E10.65
	Type 1 diabetes mellitus	E10.9
	DKA (diabetic ketoacidoses)	E11.10
Obesity	Morbid obesity due to excess calories	E66.01
	Overweight(278.02)	E66.3
	Obesity	E66.9
	BMI (body mass index), pediatric, greater than 99% for age	Z68.54

**Respiratory disease**		
	Bronchiolitis	J21.9
	Chronic rhinitis	J31.0
	Nasal polyp	J33.9
	Tracheobronchomalacia	J39.8
	Bronchitis	J40
	Chronic respiratory failure with hypoxia and hypercapnia	J96.11
	Chronic respiratory failure with hypoxia and hypercapnia	J96.12
	Acute on chronic respiratory failure with hypoxia and hypercapnia	J96.21
	Acute on chronic respiratory failure with hypoxia and hypercapnia	J96.22
	Respiratory failure	J96.90
	Respiratory failure with hypercapnia	J96.92
	Atelectasis of both lungs	J98.11
	Other diseases of lung, not elsewhere classified	J98.4
	Bronchopulmonary dysplasia	P27.1
	CNPAS (congenital nasal pyriform aperture stenosis)	Q30.0
	Laryngomalacia	Q31.5
	Laryngeal cleft	Q31.8
	Congenital malformation of larynx	Q31.9
	Tracheomalacia, congenital	Q32.0
	Bronchomalacia, congenital	Q32.2
	Hypoplasia of right lung	Q33.3
Asthma	Asthma exacerbation in COPD	J44.1
	Mild intermittent asthma without complication	J45.20
	Mild intermittent asthma with acute exacerbation	J45.21
	Mild intermittent asthma with status asthmaticus	J45.22
	Mild persistent asthma without complication	J45.30
	Mild persistent asthma with acute exacerbation	J45.31
	Moderate persistent asthma without complication	J45.40
	Moderate persistent asthma with exacerbation	J45.41
	Moderate persistent asthma with status asthmaticus	J45.42
	Severe persistent asthma without complication	J45.50
	Severe persistent asthma refractory to systemic steroids with acute exacerbation	J45.51
	Severe persistent asthma with status asthmaticus	J45.52
	Asthma exacerbation	J45.901
	Status asthmaticus	J45.902
	Asthma	J45.909
	Asthma in remission	J45.998
	Severe persistent asthma refractory to systemic steroids with acute exacerbation	Z79.52
	History of asthma	Z87.09
**Neurological diseases**		
	Focal epilepsy	G40.109
	Generalized epilepsy	G40.309

Epileptic encephalopathy	G40.409
Childhood onset epileptic encephalopathy	G40.802
Status epilepticus	G40.901
Epileptic encephalopathy	G93.49
Febrile seizures	R56.00
Complex febrile seizure	R56.01
Seizures, post-traumatic	R56.1
Seizure in pediatric patient	R56.9
Meningitis	G03.9
Cerebral ventriculitis	G04.90
Dural venous sinus thrombosis	G08
Friedreich's ataxia	G11.1
Bulbar weakness	G12.29
Restless leg syndrome	G25.81
Extrapyramidal disease and abnormal movement disorder	G25.9
Infantile spasms	G40.822
Seizure disorder	G40.909
Intractable seizure disorder	G40.919
Absence seizure	G40.A09
Migraines	G43.909
New daily persistent headache	G44.52
Other headache syndrome	G44.89
Insomnia	G47.00
Trouble getting to sleep	G47.09
Sleep disorder, circadian, irregular sleep-wake type	G47.23
Sleep disorder breathing	G47.30
Obstructive sleep apnea (adult) (pediatric)	G47.33
Sleep disturbance	G47.9
Drug-induced polyneuropathy	G62.0
Spastic quadriplegic cerebral palsy	G80.0
Spastic diplegia	G80.1
Congenital quadriplegia	G80.8
Cerebral palsy	G80.9
Spastic quadriplegia	G82.50
Post-op pain	G89.18
Obstructive hydrocephalus	G91.1
Hydrocephalus	G91.9
Increased intracranial pressure	G93.2
Acute metabolic encephalopathy	G93.41
Chiari I malformation	G93.5
Syrinx of spinal cord	G95.0
CSF leak	G96.0
Post lumbar puncture headache	G97.1

	Microcephaly	Q02
	Aqueductal stenosis	Q03.0
	Hydrocephalus associated with congenital aqueduct stenosis	Q03.9
	Absent corpus callosum	Q04.0
	HPE (holoprosencephaly)	Q04.2
	Congenital reduction deformities of brain	Q04.3
	Porencephalic cyst, congenital	Q04.6
	Absent septum pellucidum	Q04.8
	Tethered cord	Q06.8
**Psychiatric disorders**		
	Mood disorder with depressive features due to medical condition	F06.31
	Sedative withdrawal without complication	F13.230
	Undifferentiated schizophrenia	F20.3
	Psychosis	F29
	Bipolar affective disorder	F31.9
	MDD (major depressive disorder), single episode, mild	F32.0
	Current moderate episode of major depressive disorder without prior episode	F32.1
	Major depressive disorder, single episode, severe without psychotic features	F32.2
	Current severe episode of major depressive disorder with psychotic features without prior episode	F32.3
	Depression	F32.9
	Moderate episode of recurrent major depressive disorder	F33.1
	Major depressive disorder, recurrent severe without psychotic features	F33.2
	MDD (major depressive disorder), recurrent, severe, with psychosis	F33.3
	Recurrent major depressive disorder	F33.9
	DMDD (disruptive mood dysregulation disorder)	F34.81
	Mood disorder	F39
	Social anxiety disorder	F40.10
	Panic anxiety syndrome	F41.0
	GAD (generalized anxiety disorder)	F41.1
	Other specified anxiety disorders	F41.8
	Anxiety	F41.9
	OCD (obsessive compulsive disorder)	F42.9
	Post traumatic stress disorder (PTSD)	F43.10
	Chronic post-traumatic stress disorder (PTSD)	F43.12
	Adjustment disorder with depressed mood	F43.21
	Adjustment disorder with anxious mood	F43.22
	Adjustment disorder with mixed anxiety and depressed mood	F43.23
	Adjustment disorder with disturbance of conduct	F43.24
	Mixed disturbance of emotions and conduct as adjustment reaction	F43.25
	Trauma and stressor-related disorder	F43.9
	Mental health problem	F48.9
	Avoidant and restrictive food intake disorder	F50.82
	Self induced vomiting	F50.89

	Psychological factors affecting medical condition	F54
	Borderline personality disorder	F60.3
	Impulse control disorder	F63.9
	Gender dysphoria in adolescent and adult	F64.0
	Psychiatric diagnosis	F99
**Neurological diseases**		
	Moderate intellectual disability	F71
	Severe mental retardation	F72
	Intellectual disability	F79
	Language delay	F80.1
	Mixed receptive-expressive language disorder	F80.2
	Social communication disorder, pragmatic	F80.82
	Other developmental speech or language disorder	F80.89
	Speech delay	F80.9
	Developmental non-verbal disorder	F81.89
	Learning disorder	F81.9
	Autism	F84.0
	Sensory integration disorder	F88
	Disorder of psychological development	F89
	ADHD (attention deficit hyperactivity disorder), predominantly hyperactive impulsive type	F90.1
	Attention deficit hyperactivity disorder (ADHD), combined type	F90.2
	Hyperkinesis of childhood with developmental delay	F90.8
	ADHD	F90.9
	Oppositional defiant disorder	F91.3
	Temper tantrums	F91.8
	Separation anxiety disorder	F93.0
	Reactive attachment disorder	F94.1
	Motor tic disorder	F95.8
	Developmental feeding disorder	F98.29
	Head banging	F98.4
	ADD (attention deficit disorder)	F98.8
	Behavioral disorder in pediatric patient	F98.9
	Fetal alcohol syndrome	Q86.0
	Pierre Robin syndrome	Q87.0
	Beckwith-Wiedemann syndrome	Q87.3
	Cayler syndrome	Q87.89
	Dysmorphic features	Q89.7
	Congenital anomaly	Q89.9
	Down syndrome	Q90.9
	Trisomy 9	Q92.8
	Cri-du-chat syndrome	Q93.4
	Chromosome 1p36 deletion syndrome	Q93.59
	Chromosome 1q21.1 microdeletion syndrome	Q93.88

	1P36 deletion syndrome	Q93.89
	Chromosomal deletion syndrome	Q93.9
	Duplication of chromosome 6q	Q99.8
	Chromosomal abnormality	Q99.9
**Blood disorders**		
	Myelodysplastic syndrome	D46.9
	Transient myeloproliferative disorder	D47.1
	Iron deficiency anemia secondary to inadequate dietary iron intake	D50.8
	Iron deficiency anemia	D50.9
	Alpha thalassemia	D56.0
	Hemoglobin S trait with alpha thalassemia trait	D56.3
	Sickle cell disease with HPFH	D56.4
	Hemoglobin SS disease with vasoocclusive crisis	D57.00
	Acute chest syndrome	D57.01
	Hemoglobin S-S disease	D57.1
	Sickle cell trait	D57.3
	Hereditary spherocytosis	D58.0
	Hemoglobin C trait	D58.2
	HUS (hemolytic uremic syndrome)	D59.3
	Pancytopenia	D61.818
	Anemia in stage 5 chronic kidney disease	D63.1
	Anemia of infection and chronic disease	D63.8
	Antineoplastic chemotherapy induced anemia	D64.81
	Anemia due to immunosuppressive medication	D64.9
	DIC (disseminated intravascular coagulation)	D65
	Von Willebrand's disease	D68.0
	Factor XI deficiency	D68.1
	Coagulation factor disorder	D68.4
	Coagulation disorder	D68.9
	Chemotherapy-induced thrombocytopenia	D69.59
	Thrombocytopenia	D69.6
	Chemotherapy-induced neutropenia	D70.1
	Other neutropenia	D70.8
	Fever and neutropenia	D70.9
	Lymphopenia	D72.810
	Leukopenia	D72.819
	Lymphocytosis	D72.820
	Leukocytosis	D72.829
	G6PD deficiency	D75.A
**Gastrointestinal disorders**		
	Gastroesophageal reflux disease with esophagitis	K21.0
	GERD (gastroesophageal reflux disease)	K21.9
	Anastomotic ulcer	K28.9

Pyloric stenosis	K31.1
Crohn's colitis	K50.10
Crohn's disease of both small and large intestine with rectal bleeding	K50.811
Crohn's disease of small and large intestines with complication	K50.819
Crohn's disease	K50.90
Ulcerative rectosigmoiditis with rectal bleeding	K51.311
Ulcerative colitis	K51.90
Ulcerative colitis with complication	K51.919
Food protein induced enterocolitis syndrome (FPIES)	K52.21
Eosinophilic gastroenteritis	K52.81
IBD (inflammatory bowel disease)	K52.9
NEC (necrotizing enterocolitis)	K55.30
Intestinal ischemia	K55.9
Intussusception	K56.1
Fecal impaction	K56.41
Bowel obstruction	K56.609
Colonic stricture	K56.699
IBS (irritable bowel syndrome)	K58.9
Generalized intestinal dysmotility	K59.8
Abscess of anal and rectal regions	K61.2
Rectal bleeding	K62.5
Anal or rectal pain	K62.89
Spontaneous bacterial peritonitis	K65.2
Bacterial peritonitis	K65.9
Pneumoperitoneum	K66.8
Acute liver failure without hepatic coma	K72.00
Liver fibrosis	K74.0
Hepatitis	K75.9
Chronic passive hepatic congestion	K76.1
Portal hypertension	K76.6
Chronic cholecystitis with calculus	K80.10
Cholelithiasis	K80.20
Gall bladder disease	K82.9
Gall stone pancreatitis	K85.10
Drug-induced acute pancreatitis	K85.30
Pancreatitis	K85.90
PLE (protein losing enteropathy)	K90.49
Malabsorption	K90.9
Postresectional malabsorption syndrome	K91.2
Macroglossia	Q38.2
Ileal atresia	Q41.2
Hirschsprung's disease	Q43.1
Duplication, intestine	Q43.4

	Caroli disease	Q44.5
	Annular pancreas	Q45.1
**Eczema-atopic dermatitis**		
	Impetiginized atopic dermatitis	L01.1
	Atopic dermatitis	L20.9
	Contact dermatitis and other eczema, due to unspecified cause	L25.9
	Eczema	L30.9
**Prematurity**		
	ROP (retinopathy of prematurity)	H35.109
	Premature infant with gestation of 30-35 weeks	IMO0002
	Preterm delivery	O60.10X0
	Premature infant, 750-999 gm	P07.03
	Prematurity, 2,000-2,499 grams, 33-34 completed weeks	P07.18
	Extreme prematurity	P07.20
	Premature birth	P07.30
	Premature infant of 29 weeks gestation	P07.32
	Preterm newborn, gestational age 31 completed weeks	P07.34
	Premature infant of 32 weeks gestation	P07.35
**Renal disease**		
	Benign hypertension with end-stage renal disease	I12.0
	Hypertension secondary to other renal disorders	I15.1
	Glomerulonephritis due to antineutrophil cytoplasmic antibody (ANCA) positive vasculitis	I77.89
	Nephrotic syndrome with lesion of membranous glomerulonephritis	N04.2
	Nephrotic syndrome	N04.9
	FSGS (focal segmental glomerulosclerosis)	N05.1
	IgM nephropathy	N05.8
	Glomerulonephritis due to antineutrophil cytoplasmic antibody (ANCA) positive vasculitis	N05.9
	Pyelonephritis, acute	N10
	Hydronephrosis	N13.30
	Anemia in stage 5 chronic kidney disease	N18.5
	ESRD (end stage renal disease) on dialysis	N18.6
	Chronic kidney disease (CKD)	N18.9
	Renal failure	N19
	Hypertension secondary to other renal disorders	N28.89
	Kidney problem	N28.9
	Polycystic kidney disease, congenital	Q61.3
	Acute renal failure on dialysis	Z99.2

Supplementary Table S2: Characteristics of children with lab-confirmed influenza by Race.

	Black or African American				Hispanic or Latino				White or Caucasian			
	**Total**	**Not Admitted**	**Admitted**	***p-value***	**Total**	**Not Admitted**	**Admitted**	***p-value***	**Total**	**Not Admitted**	**Admitted**	***p-value***
	N=113	N=99	N=14		N=357	N=337	N=20		N=516	N=479	N=37	
**Age Years**	7.00 (2.00-12.00)	7.00 (2.00-12.00)	8.00 (2.00-11.00)	0.84	5.00 (2.00-9.00)	5.00 (2.00-9.00)	4.50 (0.50-11.00)	0.44	7.00 (3.00-12.00)	7.00 (3.00-12.00)	6.00 (3.00-12.00)	0.59
**Patient Sex**				0.79				0.67				0.26
**Female**	44 (38.9%)	39 (39.4%)	5 (35.7%)		159 (44.5%)	151 (44.8%)	8 (40.0%)		227 (44.0%)	214 (44.7%)	13 (35.1%)	
**Male**	69 (61.1%)	60 (60.6%)	9 (64.3%)		198 (55.5%)	186 (55.2%)	12 (60.0%)		289 (56.0%)	265 (55.3%)	24 (64.9%)	
**Comorbidities**												
**At least one Comorbidity**	64 (56.6%)	53 (53.5%)	11 (78.6%)	0.077	198 (55.5%)	182 (54.0%)	16 (80.0%)	**0.023**	195 (37.8%)	163 (34.0%)	32 (86.5%)	**<0.001**
**Heart disease**	6 (5.3%)	4 (4.0%)	2 (14.3%)	0.11	7 (2.0%)	6 (1.8%)	1 (5.0%)	0.31	13 (2.5%)	10 (2.1%)	3 (8.1%)	**0.024**
**Malignancy**	1 (0.9%)	0 (0.0%)	1 (7.1%)	**0.008**	7 (2.0%)	6 (1.8%)	1 (5.0%)	0.31	4 (0.8%)	3 (0.6%)	1 (2.7%)	0.17
**Diabetes**	1 (0.9%)	1 (1.0%)	0 (0.0%)	0.71	4 (1.1%)	2 (0.6%)	2 (10.0%)	**<0.001**	9 (1.7%)	5 (1.0%)	4 (10.8%)	**<0.001**
**Obesity**	0 (0.0%)	0 (0.0%)	0 (0.0%)		10 (2.8%)	9 (2.7%)	1 (5.0%)	0.54	3 (0.6%)	3 (0.6%)	0 (0.0%)	0.63
**Endocrine or metabolic disease**	9 (8.0%)	6 (6.1%)	3 (21.4%)	**0.047**	20 (5.6%)	16 (4.7%)	4 (20.0%)	**0.004**	23 (4.5%)	11 (2.3%)	12 (32.4%)	**<0.001**
**Asthma**	34 (30.1%)	31 (31.3%)	3 (21.4%)	0.45	94 (26.3%)	91 (27.0%)	3 (15.0%)	0.24	63 (12.2%)	58 (12.1%)	5 (13.5%)	0.80
**Respiratory disease**	4 (3.5%)	3 (3.0%)	1 (7.1%)	0.44	24 (6.7%)	22 (6.5%)	2 (10.0%)	0.55	25 (4.8%)	20 (4.2%)	5 (13.5%)	**0.011**
**Neurological disease**	15 (13.3%)	8 (8.1%)	7 (50.0%)	**<0.001**	29 (8.1%)	25 (7.4%)	4 (20.0%)	**0.045**	32 (6.2%)	22 (4.6%)	10 (27.0%)	**<0.001**
**Psychiatric disorders**	6 (5.3%)	3 (3.0%)	3 (21.4%)	**0.004**	17 (4.8%)	15 (4.5%)	2 (10.0%)	0.26	34 (6.6%)	28 (5.8%)	6 (16.2%)	**0.014**
**Neurodevelopment disorders**	12 (10.6%)	10 (10.1%)	2 (14.3%)	0.63	43 (12.0%)	40 (11.9%)	3 (15.0%)	0.68	58 (11.2%)	46 (9.6%)	12 (32.4%)	**<0.001**
**Blood disorders**	11 (9.7%)	9 (9.1%)	2 (14.3%)	0.54	20 (5.6%)	17 (5.0%)	3 (15.0%)	**0.060**	15 (2.9%)	9 (1.9%)	6 (16.2%)	**<0.001**
**Gastrointestinal disorders**	12 (10.6%)	8 (8.1%)	4 (28.6%)	**0.020**	26 (7.3%)	20 (5.9%)	6 (30.0%)	**<0.001**	45 (8.7%)	37 (7.7%)	8 (21.6%)	**0.004**
**Eczema-atopic dermatitis**	15 (13.3%)	12 (12.1%)	3 (21.4%)	0.34	42 (11.8%)	42 (12.5%)	0 (0.0%)	0.093	19 (3.7%)	15 (3.1%)	4 (10.8%)	**0.017**
**Prematurity**	7 (6.2%)	6 (6.1%)	1 (7.1%)	0.88	21 (5.9%)	18 (5.3%)	3 (15.0%)	0.074	11 (2.1%)	11 (2.3%)	0 (0.0%)	0.35
**Renal disease**	1 (0.9%)	0 (0.0%)	1 (7.1%)	**0.008**	6 (1.7%)	6 (1.8%)	0 (0.0%)	0.55	6 (1.2%)	5 (1.0%)	1 (2.7%)	0.36
